# GalNAc/Gal-Binding *Rhizoctonia solani* Agglutinin Has Antiproliferative Activity in *Drosophila melanogaster* S2 Cells via MAPK and JAK/STAT Signaling

**DOI:** 10.1371/journal.pone.0033680

**Published:** 2012-04-18

**Authors:** Mohamad Hamshou, Els J. M. Van Damme, Gianni Vandenborre, Bart Ghesquière, Geert Trooskens, Kris Gevaert, Guy Smagghe

**Affiliations:** 1 Laboratory of Agrozoology, Department of Crop Protection, Faculty of Bioscience Engineering, Ghent University, Ghent, Belgium; 2 Laboratory of Biochemistry and Glycobiology, Department of Molecular Biotechnology, Faculty of Bioscience Engineering, Ghent University, Ghent, Belgium; 3 Department of Medical Protein Research, VIB, Ghent, Belgium; 4 Department of Biochemistry, Ghent University, Ghent, Belgium; 5 Department of Mathematical Modelling, Statistics and Bioinformatics, Faculty of Bioscience Engineering, Ghent University, Ghent, Belgium; St. Georges University of London, United Kingdom

## Abstract

*Rhizoctonia solani* agglutinin, further referred to as RSA, is a lectin isolated from the plant pathogenic fungus *Rhizoctonia solani*. Previously, we reported a high entomotoxic activity of RSA towards the cotton leafworm *Spodoptera littoralis*. To better understand the mechanism of action of RSA, *Drosophila melanogaster* Schneider S2 cells were treated with different concentrations of the lectin and FITC-labeled RSA binding was examined using confocal fluorescence microscopy. RSA has antiproliferative activity with a median effect concentration (EC_50_) of 0.35 µM. In addition, the lectin was typically bound to the cell surface but not internalized. In contrast, the N-acetylglucosamine-binding lectin WGA and the galactose-binding lectin PNA, which were both also inhibitory for S2 cell proliferation, were internalized whereas the mannose-binding lectin GNA did not show any activity on these cells, although it was internalized. Extracted DNA and nuclei from S2 cells treated with RSA were not different from untreated cells, confirming inhibition of proliferation without apoptosis. Pre-incubation of RSA with *N*-acetylgalactosamine clearly inhibited the antiproliferative activity by RSA in S2 cells, demonstrating the importance of carbohydrate binding. Similarly, the use of MEK and JAK inhibitors reduced the activity of RSA. Finally, RSA affinity chromatography of membrane proteins from S2 cells allowed the identification of several cell surface receptors involved in both signaling transduction pathways.

## Introduction

Lectins are a diverse group of proteins or glycoproteins that can bind to carbohydrates on the cell surface and induce various biological effects. They are omnipresent in nature, and are found in plants, animals and microorganisms. In addition to the extensive studies on plant lectins, a number of carbohydrate-binding proteins has been isolated from fungi, especially mushrooms, and studied for their physiological functions. Fungal lectins probably play an important role in some biological processes such as dormancy, growth and morphogenesis [1], [2]. At present very few fungal lectins have been studied with regard to their insecticidal activity.

The *Rhizoctonia solani* agglutinin (RSA) is a fungal lectin which was purified from the sclerotes of the soil plant pathogenic fungus *Rhizoctonia solani* [3]. RSA is a homodimeric protein consisting of two 15.5 kDa subunits with specificity towards *N*-acetylgalactosamine (GalNAc) and galactose [4], and has been proposed to play a role as a storage protein in the sclerotes of the fungus [5], [6]. Previously, we reported that RSA has toxic effects on the growth, development and survival of the cotton leafworm *Spodoptera littoralis* which is an important pest in agriculture [7].

Many factors can influence the biological activity of lectins on cells such as their binding on the cell surface or internalization in the cell and the availability of suitable targets. It was shown that the fungal lectin from *Xerocomus chrysenteron* (XCL), which exerts high toxicity in several insect species from different orders [8], [9], is internalized by clathrin-dependent endocytosis and is then delivered to late endosome/lysosome compartments in insect (SF9) or mammalian (NIH-3T3 and Hela) cell lines [10]. The internalization of the *Sambucus nigra* agglutinin (SNA-I) which induces (cyto)toxicity by caspase-dependent apoptosis, occurs via clathrin and caveolae-mediated endocytosis in insect midgut CF-203 cells [11], [12]. In contrast, other cytotoxic lectins bind to the cell surface and cause cell death without internalization of the lectin into the cytoplasm. For example, the fungal lectin from *Sclerotinia sclerotiorum* (SSA) with a carbohydrate specificity for galactose (Gal) and *N*-acetylgalactosamine (GalNAc), was found to be toxic to midgut CF-203 cells, although it was not taken up in the cells but only bound to the cell surface [13]. SSA was proposed to kill the cells by the induction of apoptosis via a caspase-3 independent pathway. The *Cucumaria echinata* lectin (CEL-I) also bound to the cell surface and exerted high toxicity towards mammalian cells [14], but the effect was apoptosis-independent by causing changes in the plasma membrane integrity.

In the present study, the mode of action of RSA was investigated in the *Drosophila melanogaster* Schneider S2 cell line. This cell line was originally derived from primary cultures of late-stage of *D. melanogaster* embryos [15]. These cells are typically round with a diameter of 15–20 µm and many features of the S2 cell line suggest that it is derived from a macrophage-like lineage. For this study the S2 cells were chosen because *D. melanogaster* represents an important model insect and because of the availability of the *Drosophila* genome and proteome database [16]. In addition, a comparative analysis was made of the activity of RSA and selected plant lectins in S2 cells, and we investigated to what extent the FITC-labeled lectins were bound and/or taken up by these insect cells. For RSA the importance of its binding to carbohydrates on the cell surface was shown using an excess of GalNAc in the culture medium. In addition, nuclear morphological changes and DNA fragmentation were evaluated in RSA-treated S2 cells to study whether RSA activity relates to apoptosis. Different kinase inhibitors were used on S2 cells to block specific signaling transduction pathways, and highlighted those that were involved in the RSA signal transduction pathway leading to inhibition of cell proliferation. Finally, potential target proteins for RSA in the cell membrane of S2 cells were identified using RSA affinity chromatography and LC-MS/MS.

## Materials and Methods

### Isolation of Lectins and Labeling with FITC

RSA was isolated from the sclerotes of the plant pathogenic fungus *R. solani* using affinity chromatography on galactose-Sepharose 4B and ion exchange chromatography on Q Fast Flow column (GE Healthcare, Uppsala, Sweden), as described previously [13]. Other plant lectins used in this study were peanut (*Arachis hypogaea*) agglutinin (PNA), wheat germ (*Triticum aestivum*) agglutinin (WGA) and snowdrop (*Galanthus nivalis*) lectin (GNA) recognizing Gal, *N*-acetylglucosamine (GlcNAc) and mannose, respectively. All these lectins were purified in the laboratory as described previously [17].

Lectins were labeled with fluorescein isothiocyanate (FITC) as described previously [13]. Briefly, lectins were dissolved in 50 mM sodium borate buffer (pH 8.5) at ratio (1mg/100 µl) and mixed with 24-fold molar excess FITC dissolved in dimethylformamide (Invitrogen, Molecular Probes, Belgium). After 1 h incubation at room temperature in the dark, the free label was removed by gel filtration on a Sephadex G25 column, equilibrated with PBS. Lectin activity in the eluted fractions was checked using an agglutination assay.

### Cell Proliferation Assay

The cytotoxic action of RSA was investigated in S2 cells, a cell line derived from *D. melanogaster* embryos (originally from The Drosophila Genomics Resource Center, Indiana University, Bloomington, IN) which was cultured in HYQ SFX-Insect medium (Perbio Science, Erembodegem, Belgium) [18]. 100 µl of a cell suspension containing 1×10^6^ cells per ml was incubated in wells of a 96-well microtiter plate for 4 days at 27°C with different concentrations of RSA or an equal amount of PBS in the control treatment. Four replicates were performed for each concentration, and the overall experiment was repeated twice. After incubation, cell proliferation was monitored using the 3-(4,5)dimethylthiazol-2-yl)-2,5-diphenyltetrazolium bromide (MTT) assay as described previously [13].

In addition, the effect of three plant lectins PNA, WGA and GNA on S2 cells was investigated and compared with that of RSA to check whether there is a correlation between carbohydrate specificity of the lectins and their antiproliferative activity on S2 cells. For each lectin, S2 cells were treated with a 0.7 µM solution of these lectins.

Detailed studies of the carbohydrate-binding properties of RSA using glycan array analyses from the Consortium for Functional Glycomics (http://www.functionalglycomics.org/glycomics/publicdata/primaryscreen.jsp) have shown that RSA interacts with GalNAc α1,3 Gal and has a clear preference for GalNAc residues over Gal. In contrast, PNA interacts well with Gal β1, 3 GalNAc, and clearly prefers Gal over GalNAc, whereas WGA interacts preferentially with GlcNAc oligomers and GNA with terminal mannose residues.

Significant differences between treatments were determined by one-way analysis of variance (ANOVA) using a *post hoc* Tukey-Kramer test which was performed using SPSS v17.0 (SPSS Inc., Chicago, IL). In addition, a concentration-response curve, a 50%-effect concentration (EC_50_) and the corresponding 95% confidence limits (95% CL) were estimated with Prism v4 (GraphPad, La Jolla, CA); the accuracy of data fitting to the sigmoid curve model was evaluated through examination of R^2^ values and the comparison of EC_50_ was done using the overlapping of 95% CL as a criterion.

### Effect of Carbohydrates on RSA Antiproliferative Activity on S2 Cells

The effect of sugars on the antiproliferative activity induced by RSA in S2 cells was investigated. Therefore, 0.7 µM of RSA was pre-incubated for one hour with the specific sugar GalNAc at 100 mM, while the non-specific sugar mannose at 100 mM was used as a negative control. Afterwards, the mixture was added to S2 cells and incubated for 24 h at 27°C. The MTT assay was used to determine cell proliferation parameters.

### RSA Activity in S2 Cells Following Pre-incubation with Kinase Inhibitors

To study the effect of kinase inhibitors on the antiproliferative activity of RSA, S2 cells were pre-incubated with different inhibitors: 10 µM of SB203580 (p38 MAP kinase inhibitor), 50 µM of PD98059 (MAP kinase (MEK) inhibitor) and 50 µM of AG490 (JAK inhibitor). All inhibitors were purchased from Calbiochem (Darmstadt, Germany). The inhibitors were used at the highest possible concentration that did not affect growth of S2 cells as determined in preliminary experiments using MTT cell viability bioassays (data not shown). After incubation in the presence or absence of the individual inhibitors for 1 h, cells were treated with 0.3 µM RSA and incubated for 3 h at 27°C. Similar volumes of solvent (DMSO for the inhibitors, and PBS for RSA) were used in all treatments as well in control cells. For every treatment, three replicates were prepared and the experiment was repeated twice. After incubation, the cell proliferation was determined using an MTT assay.

### Internalization Assay

Uptake of RSA in S2 cells was investigated as described previously [13]. In brief, cells grown on poly-L-lysine coated slides were treated with 0.7 µM of FITC-labeled RSA. Then, the cells were washed and fixed with 2% paraformaldehyde. The uptake of RSA in S2 cells was also compared to that of the plant lectins PNA, WGA and GNA. Cells were examined with a Nikon A1R confocal fluorescence microscope (Nikon, France).

### DNA Fragmentation Analysis in S2 Cells

DNA was extracted from embryonic S2 cells after 24 h of incubation in the presence or absence of RSA using a phenol/chloroform/isoamyl alcohol method as described in [11]. Then 10 µg DNA was analyzed on a 2% agarose gel and DNA was visualized by ethidium bromide staining and subsequent UV illumination.

### Nuclear Staining with Hoechst Dyes

S2 cells grown on poly-L-lysine coated glass were incubated with 0.7 µM of RSA for 24 h at 27°C. The cells were washed with PBS and fixed with 2% paraformaldehyde for 20 min. After washing with PBS, cell nuclei were stained with Hoechst/PBS (1/1000, v/v) for 15 min. After washing with PBS, slides were mounted with Vectashield (Vector Labs) and covered with a cover glass. The cells were visualized under a Nikon T*i* fluorescence microscope (Nikon Benelux) using a 60X oil immersion lens and the appropriate filters to visualize DAPI.

### Proteomic Analysis of the RSA Binding Proteins in the Membrane of S2 Cells

S2 cells grown in HYQ SFX-Insect medium were collected and washed with PBS. The cells were suspended in 2 mM of phenylmethylsulphonyl fluoride (PMSF), vortexed and frozen at −80°C overnight. The next day, cells were thawed at 4°C and the resulting solution was homogenized in an Eppendorf tube using a pestle. After vortexing for 1–2 min, the extract was centrifuged at 16,000 g for 2 h at 4°C. The supernatant was removed and the pellet was resuspended in 10 mM HEPES buffer (pH 7.4), containing 1.5% Triton X-100 and incubated at 4°C for 1 h. In between the sample was vortexed a few times. After that, the supernatant containing the membrane proteins was collected by centrifugation at 20,000 g for 30 min at 4°C and frozen at –80°C.

RSA affinity chromatography was performed as described previously [19], [20]. Briefly, membrane protein fractions were loaded on the RSA column and the captured proteins were eluted using 20 mM unbuffered 1,3-diaminopropane. The membrane glycoproteins captured by RSA were dried and re-dissolved in 50 mM triethylammoniumbicarbonate (TEAB, Sigma-Aldrich, Steinheim, Germany) pH 7.8. Following a denaturing step at 95°C for 10 min and cooling down on ice, trypsin (ultragrade, Promega, Madison, WI) was added in a 1∶100 ratio (w/w) and digestion of the protein samples was carried out overnight at 37°C. Following digestion, each protein digestion mixture (equivalent to approximately 300 µg of proteins) was acidified with 10% trifluoroacetic acid to a final concentration of 0.5%, and loaded for RP-HPLC separation on a 2.1 mm internal diameter × 150 mm 300SB-C18 column (Zorbax®, Agilent technologies, Waldbronn, Germany) using an Agilent 1100 Series HPLC system. Briefly, following a 10 min wash with 0.1% TFA in water/acetonitrile (98/2 (v/v), both Baker HPLC analysed, Mallinckrodt Baker B.V., Deventer, the Netherlands), a linear gradient to 0.1% TFA in water/acetonitrile (30/70, v/v) was applied over 100 min at a constant flow rate of 80 µl/min. 48 fractions of eluting peptides were collected between 20 and 68 min, and fractions separated by 16 min were pooled, dried and stored at −20°C until LC-MS/MS analysis.

These dried fractions (16 fraction per sample) were re-dissolved in 80 µl of 2.5% acetonitrile and 8 µl was used for LC-MS/MS analysis using an Ultimate 3000 HPLC system (Dionex, Amsterdam, the Netherlands) in-line connected to a LTQ Orbitrap XL mass spectrometer (Thermo Electron, Bremen, Germany). Peptides were first trapped on a trapping column (PepMap™ C18 column, 0.3 mm I.D. ×5 mm (Dionex)) and, following back-flushing from this trapping column, peptides were loaded on a 75 µm I.D. ×150 mm reverse-phase column (PepMap™ C18, Dionex). Bound peptides were eluted with a linear gradient of 1.8% solvent B (0.05% formic acid in water/acetonitrile (2/8, v/v)) increase per minute at a constant flow rate of 300 nl/min.

The mass spectrometer was operated in data-dependent mode, and automatically switched between MS and MS/MS acquisition for the six most abundant ion peaks per MS spectrum. Full scan MS spectra were acquired at a target value of 1E6 with a resolution of 30,000. The six most intense ions were then isolated for fragmentation in the linear ion trap. In this LTQ, MS/MS scans were recorded in profile mode at a target value of 5,000. Peptides were fragmented after filling the ion trap with a maximum ion time of 10 ms and a maximum of 1E4 ion counts. From the MS/MS data in each LC-run, Mascot generic files (mgf) were created using the Mascot Distiller software (version 2.2.1.0, Matrix Science). When generating these peak lists, grouping of spectra was performed with a maximum intermediate retention time of 30 s and maximum intermediate scan count of 5, where possible. Grouping was done with 0.1 Da tolerance on the precursor ion. A peak list was only generated when the MS/MS spectrum contained more than 10 peaks, no de-isotoping was performed and the relative S/N (signal/noise) limit was set at 2.

The generated peak lists were searched with Mascot using the Mascot Daemon interface (version 2.3.01, Matrix Science). Spectra were searched against the UniProt database (http://www.uniprot.org/) with taxonomy restricted to *Drosophila melanogaster*. Variable modifications were set to methionine oxidation, pyro-glutamate formation of amino terminal glutamine and acetylation of the N-terminus. Mass tolerance of the precursor ions was set to 10 ppm and for fragment ions to 0.5 Da. The peptide charge was set to 1+, 2+ or 3+ and one missed tryptic cleavage site was allowed. Also, Mascot’s C13 setting was set to 1. Only peptides that were ranked one and scored above the threshold score set at 99% confidence were withheld. For extraction and storage of peptide identifications, the ms_lims platform was used (ref: PMID: 20058248). All data were submitted to PRIDE (login: …; password:…).

The identified proteins were annotated by performing a BLAST search (http://blast.ncbi.nlm.nih.gov) against GenBank. In addition, the number of predicted *N*-glycosylation sites present on the polypeptide backbone was calculated using the NetNGlyc 1.0 server (http://www.cbs.dtu.dk/services/NetNGlyc). Only Asn-X-Ser/Thr sequences (where X is any amino acid except proline) with a prediction score >0.5 were withheld as potential N-glycosylation sites. The potential *O*-glycosylation sites were predicted using NetOGlyc 3.1 Server (http://www.cbs.dtu.dk/services/NetOGlyc-3.1/) and only sequences with a prediction score >0.5 were withheld as potential *O*-glycosylation sites. Finally, the location or orientation of the predicted glycoproteins in the cell membrane was determined using the TMHMM Server v. 2.0 server (http://www.cbs.dtu.dk/services/TMHMM/).

**Figure 1:**
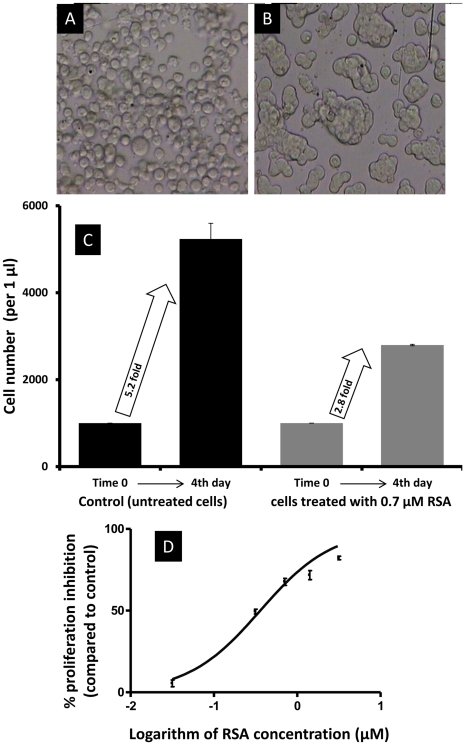
Effect of RSA on S2 cells. (A) Control, (B) Treated cells with 0.7 µM RSA, (C) S2 cell number at time zero (at the beginning of the assay) and after 4 days incubation in the presence and absence of 0.7 µM RSA. Cell numbers increased 5.2 and 2.8 fold in control and treated cells, respectively, which showed clearly that RSA inhibited cell proliferation. (D) Concentration-response curves of S2 cells challenged with RSA for 4 days after sigmoid curve fitting in Prism v4. Cell number was measured using an MTT assay.

**Figure 2:**
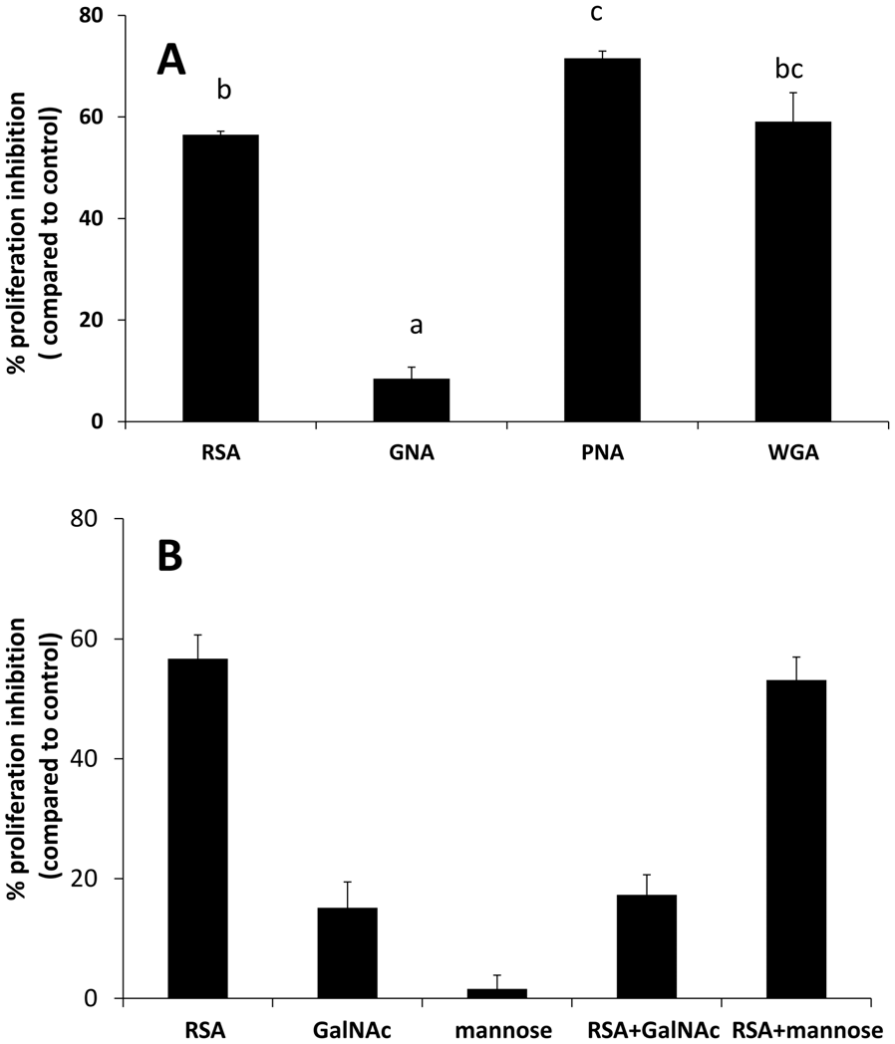
Effect by lectins on cell proliferation in *Drosophila melanogaster* S2 cells. (A) Effect by RSA, GNA, PNA and WGA when cells were treated with lectin at 0.7 µM for 24 h. (B) Inhibitory effect of sugars on the activity of RSA on S2 cells. 0.7 µM RSA was pre-incubated with 100 µM of the specific sugar GalNAc or the non-specific sugar mannose (negative control) and PBS in the control treatments for 1 h. Then the mixtures of RSA and sugars were added to S2 cells and incubated for 24 h at 27°C. Cell proliferation was measured using an MTT assay. Data are presented as mean percentages of cell proliferation inhibition ± SE compared to the control, and based on four repeats. Values are followed by a different letter (a-c) are significantly different (*post hoc* Tukey-Kramer test with p = 0.05).

**Figure 3:**
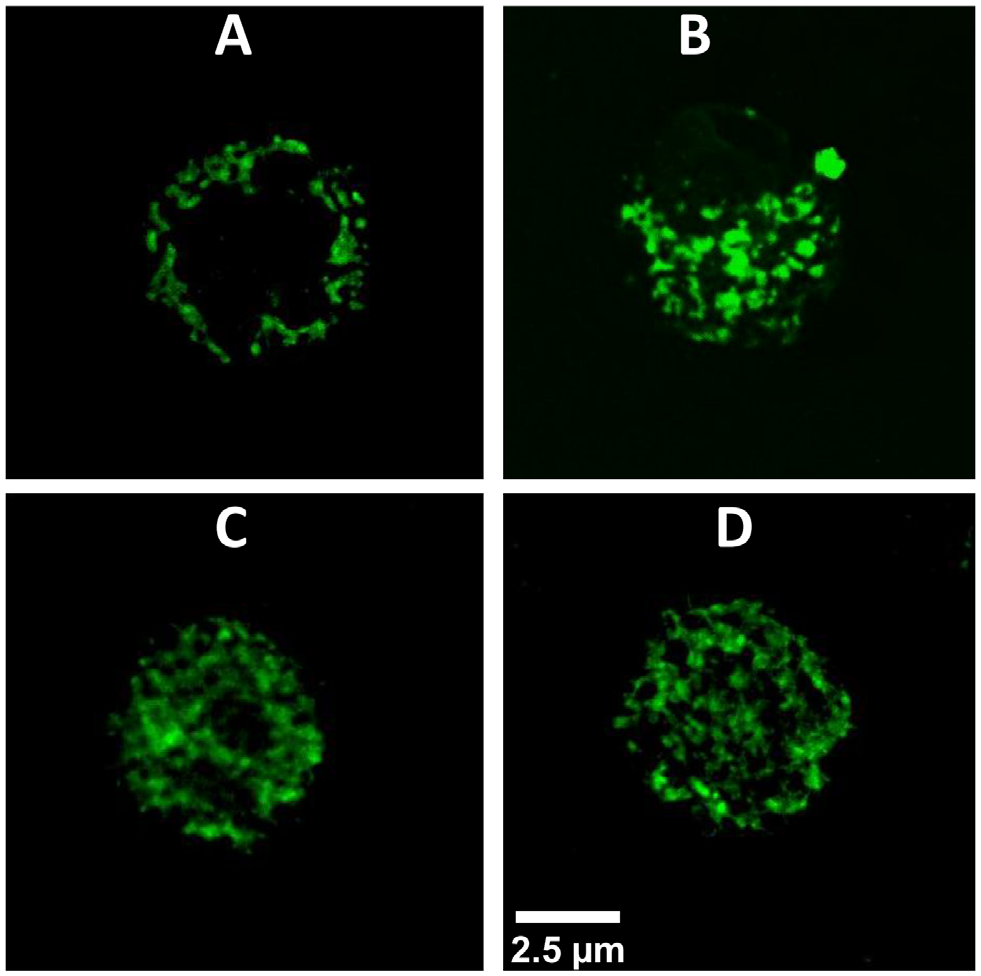
Binding and uptake of lectins in *Drosophila melanogaster* S2 cells. Confocal microscopy S2 cells incubated with different lectins: S2 cells were incubated with 0.7 µM FITC-lectin for 1 h. (A) RSA- FITC (B) GNA-FITC (C) WGA-FITC and (D) PNA-FITC. Scale bars are 2.5 µM.

**Figure 4:**
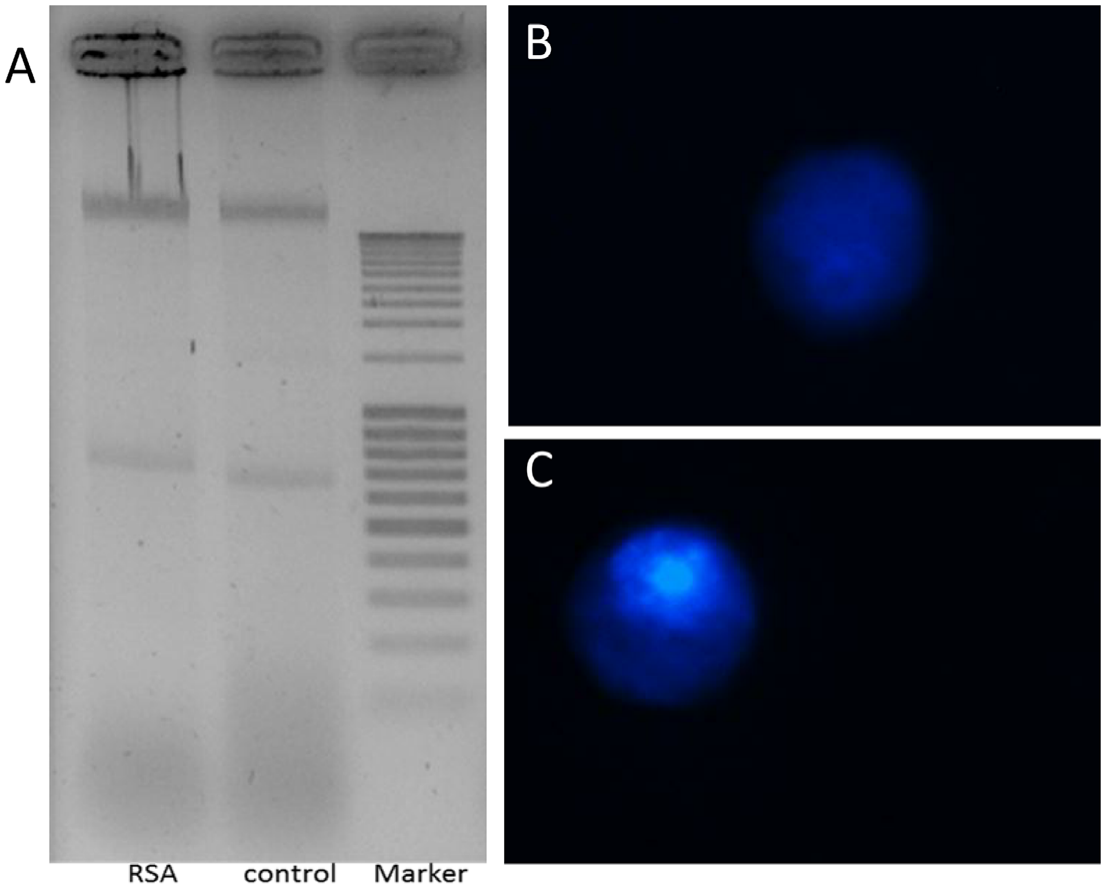
Effect of RSA to induce apoptosis in *Drosophila melanogaster* S2 cells. (A) DNA fragmentation in S2 cells. Cells were treated with 0.7 µM RSA compared to control (untreated) cells. Ten micrograms of extracted DNA was loaded on the 2% agarose gel. (B,C) Nuclear condensation assay: Upon treatment with 0.7 µM RSA, the nuclei of the S2 cells were stained with Hoechst. Typically, treated cells (C) showed a normal, non-fragmented nucleus similar to the untreated control cells (B).

**Figure 5:**
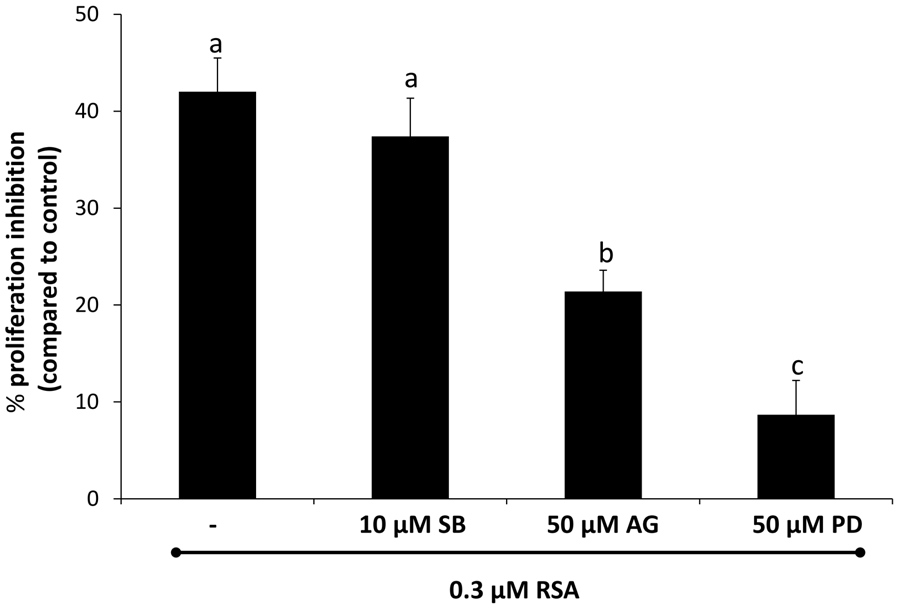
Inhibition of RSA activity after pre-incubation of the S2 cells with different kinase inhibitors. Cells were pre-incubated with three inhibitors (individually): 10 µM of SB203580 (p38 MAP kinase inhibitor), 50 µM of PD98059 [MAP kinase, (MEK) inhibitor] and 50 µM of AG490 (JAK inhibitor) for 1 h before exposure to 0.3 µM RSA for 24 h. Values are given as means ± SEM based on two independent repetitions. Values are followed by a different letter (a-c) are significantly different (*post hoc* Tukey-Kramer test with p = 0.05).

**Table 1:** List of putatively glycosylated membrane proteins with *N-* and/or *O*-glycans located on the cell surface, identified after RSA affinity chromatography and LC-MS/MS analysis.

Accession	Protein name
A1Z7G7	Latrophilin Cirl
O44386	Integrin α-PS3
P10040	Protein crumbs
P20241	Neuroglian
P42787	Carboxypeptidase D
P91660	Probable multidrug resistance-associated protein lethal(2)03659
Q27591	Integrin β-nu
Q29BL9	LMBR1 domain-containing protein 2 homolog
Q7JW12	Thioredoxin-related transmembrane protein 2 homolog
Q95TU8	Netrin receptor unc-5
Q9V4A7	Plexin-B
Q9V7H4	Transmembrane protein 131 homolog
Q9VAF0	Uncharacterized protein CG7816
Q9VLM5	Dolichyl-diphosphooligosaccharide-protein glycosyltransferase subunit DAD1
Q9VQH2	Dual oxidase
Q9VRJ2	FIT family protein CG10671
Q8MSU3	Putative ferric-chelate reductase 1 homolog

## Results

### RSA Causes Inhibition of Cell Proliferation in S2 Cells

Exposure of S2 cells to different concentrations of RSA for 4 days caused an inhibitory effect on cell proliferation. The numbers of S2 cells increased 5.2 fold in the control series, but only 2.8 fold in cells exposed to 0.7 µM RSA (Fig. 1C). The latter cells also showed typical clumping (Fig. 1B). As depicted in Fig. 1D, sigmoid curve analysis estimated a 50%-response concentration (EC_50_) for RSA of 0.35 µM (95% CL: 0.32–0.41; R^2^ = 0.9).

In a separate experiment S2 cells were treated with RSA or with selected plant lectins with different carbohydrate binding specificities. A comparative analysis was made for S2 cells exposed to 0.7 µM of RSA, PNA, WGA and GNA for 24 h. While the cellular proliferation inhibitory effects by RSA (56±1%) and WGA (59±5%) were very similar (p = 0.84), PNA showed a higher inhibition (71±1%; p = 0.03) whereas GNA caused no effect (Fig. 2A).

### Importance of Carbohydrate Binding for Antiproliferative Activity of RSA

As shown in Fig. 2B, pre-incubation of RSA with 100 mM GalNAc reduced the antiproliferative activity of the lectin with about 70%, indicating that GalNAc competes with binding of RSA to the cell. In contrast, 100 mM mannose did not affect the RSA activity.

### Binding and Internalization of RSA Compared to Plant Lectins

Confocal microscopy analysis of S2 cells exposed to FITC-labeled RSA demonstrated that the fungal lectin bound to the cell surface but was not internalized (Fig. 3A). In contrast, the plant lectins GNA, WGA and PNA were clearly taken up by the S2 cells as shown in Fig. 3B, C and D.

### RSA Treatment does not Induce Apoptosis

When S2 cells were incubated with RSA at 0.7 µM for 24 h, no DNA fragmentation was observed (Fig. 4A). In addition, there were no signs of apoptosis such as condensed and fragmented nuclei or apoptotic bodies in the RSA-treated cells (Fig. 4C).

### Effect of Kinase Inhibitors on RSA Activity

Pre-incubation of S2 cells with different MEK and JAK inhibitors for 1 h significantly (p<0.0001) reduced the antiproliferative activity of RSA (Fig. 5). The inhibitors for MEK and JAK caused a respective reduction of 49±4% and 80±5%. In contrast, the p38 MAP kinase inhibitor had no effect.

### Proteomic Analysis of Membrane Proteins of S2 Cells Retained on RSA Affinity Column

Chromatography on immobilized RSA was used to capture surface glycoproteins from S2 cells. Following LC-MS/MS analysis, 4127 peptides were sequenced, leading to the identification of 216 proteins. Of these only 34 proteins were found to be cell membrane proteins and most of them (32 proteins) have putative *N*- and/or *O*-glycosylation site(s) as determined by the NetNGlyc 1.0 and NetOGlyc 3.1 algorithms. Finally, with the use of TMHMM Server, 17 proteins were predicted to have *N*- and/or *O*-glycosylation site(s) oriented towards the cell surface (Table 1; Fig. S1, S2, S3, S4), suggesting that these particular proteins could be possible binding partners for RSA.

## Discussion

Previously, we have investigated the insecticidal activity of RSA towards the cotton leafworm *S. littoralis* [7]. We demonstrated that RSA has high entomotoxic activity on the development and survival of this economically important caterpillar insect. Therefore RSA has been reported as an insecticidal protein that could possibly be used in crop protection [7]. At present, the mechanism behind the toxic effect of RSA is not known. In this paper, an attempt was made to elucidate the mode of action of this lectin with the use of S2 cells derived from embryos of *D. melanogaster*.

The exposure of S2 cells to RSA resulted in a significant reduction of cell proliferation. Cell trafficking with FITC-labeled RSA under a confocal microscope demonstrated that the lectin was not taken up by the S2 cells, but bound to their cell surface. Since RSA preferentially binds to GalNAc (and to a lesser extent Gal), which effectively inhibited the RSA activity in S2 cells, it appears that the binding of RSA to specific carbohydrate moieties on the cell surface is a prerequisite for its activity which initiated a cascade of signaling process(es) inside the cells leading to inhibition of cellular proliferation. The interaction of RSA in S2 cells agrees with previous work in which we reported that SSA, another fungal lectin purified from the fungus *S. sclerotiorum*, exerted a dramatic toxicity in the insect midgut cell line CF-203, and similar to RSA, SSA too was only bound to the cell surface without internalization in the cell [13]. The toxicity of SSA towards CF-203 cells was accompanied with DNA fragmentation which most likely indicates that the effect of SSA is apoptosis-dependent. Moreover, Shahidi-Noghabi *et al*. [11] reported induction of apoptosis when CF-203 cells were incubated with the NeuAc(α-2,6)Gal/GalNAc specific lectin SNA-I from elderberry *Sambucus nigra*. The involvement of apoptosis in the mechanism of cell death, induced by SSA and SNA-I, in the midgut CF-203 cells raised the question whether a similar mechanism is responsible for the activity of RSA in S2 cells, but our experiments yielded different results. Indeed, no DNA fragmentation, no nuclear condensation and no apoptotic bodies were detected in S2 cells upon incubation with RSA, and the treated cells were similar in appearance to untreated cells, indicating that a different mechanism should be involved in the activity of RSA.

In an attempt to unravel the mode of action of RSA in S2 cells, various kinase inhibitors were used in an attempt to block the antiproliferative activity of the lectin. Interestingly, the activity of RSA in S2 cells was inhibited by pre-incubation of the cells with MEK inhibitor (PD98059) and JAK inhibitor (AG490). These results provide evidence that multiple pathways could be involved in the activity of RSA.

MEK (also called MAP kinase and abbreviated MAPKK) is an upstream activator of the MAP kinase (MAPK) [21]. MAPK is a family of serine/threonine kinases that can transfer various extracellular signals such as growth factors, mitogens and stress-inducing agents to the nucleus [22]. This pathway includes several kinase proteins Raf/MEK/ERK which are mainly activated by receptor tyrosine kinases (RTKs) [23], but evidence suggests that it could also be activated by other classes of membrane receptors such as integrins [24] or G-protein-coupled receptors (GPCRs) [25]. The MAPK signaling pathway has been reported to play a role in regulating cell proliferation and cell differentiation in *Drosophila* as demonstrated genetically [26]. In *Drosophila*, a small GTPase Ras oncogene at 85D (Ras85D) is activated by different signals on the cell surface receptors [27]. The activated Ras85D initiates phosphorylation of the three MAPKs within the cascade sequentially; phl (Raf1 homologue) which phosphorylates Dsor1 (MEK homologue) which then activates Rolled (Rl) (ERK homologue) [28]–[31].

Interestingly, the proteomic analysis of this project helped to identify some membrane proteins that could play a role as cell surface receptors for RSA and that could be involved in MAPK signaling pathway. Two of these RSA-binding proteins are integrins (Integrin α-PS3 and Integrin β-nu). Figures S1 and S2 show the putative *N*- and *O*-glycosylation sites and their position in both integrins. The third RSA-binding protein is a GPCR (Latrophilin Cirl) (Fig. S3). It seems that binding of RSA to one or more of these proteins might be responsible to activate the Ras85D/phl/Dsor1/Rl signaling pathway, resulting in inhibition of cellular proliferation. This hypothesis was confirmed by pre-incubation of S2 cells with the MEK (Dsor1) inhibitor which reduced the inhibition of cell proliferation of RSA for approximately 80% (Fig. 5).

The second pathway which could be involved in the activity of RSA in S2 cells involves the Janus kinase/signal transducer and activator of transcription (JAK/STAT). Involvement of this pathway in RSA activity was confirmed by using the JAK inhibitor. This pathway is reported to be activated by different membrane receptors such as the cytokine receptors, and as demonstrated in mammals and in *Drosophila*, it mediates many biological effects, for example immune response, cell survival, proliferation, differentiation, and oncogenesis [32]. Based on the structure and the activities of these receptors, they have been divided into several families, including cytokine receptors (type I and II), TNF receptor family, chemokine receptors, TGF-β receptors and members of the immunoglobulin superfamily [33]–[35]. The JAK/STAT signaling pathway mechanism starts by binding of a ligand (such as a cytokine) to a cell surface receptor, which activates JAK. Afterwards, JAK phosphorylates STAT, which translocates into the cell nucleus and regulates the expression of specific target genes [36]. In *Drosophila*, the ligand is encoded by unpaired (upd), the receptor by Domeless (DOME), JAK by hopscotch (hop), and STAT by Stat92E (also known as marelle) [37].

Proteomic analysis also identified a membrane protein which could be a cell surface receptor for RSA and that could interact with the JAK signaling pathway. This protein is Neuroglian, and its putative *N*- and *O*-glycosylation sites and their position are given in Fig. S4. This membrane protein belongs to the immunoglobulin (Ig) superfamily receptors which are considered as a receptor for the JAK/STAT pathway as mentioned above. RSA may bind to Neuroglian and in turn activates hop (JAK) which phosphorylates Stat92E or marelle (STAT). Stat92E then moves to the nucleus and inhibits the cell proliferation. Interestingly, it has recently been reported that the binding of a fungal lectin from *Rhizoctonia bataticola* lectin (RBL) to complex sugars on the cell surface of human PBMC cells also affected cell proliferation via the MAPK (but through p38 and not MEK) and JAK/STAT signaling pathways [38]. However and in contrast to RSA in S2 cells, the RBL activity was not inhibited by GalNAc, but by some glycoproteins such as mucin, fetuin and asialofetuin. Moreover, multiple MAPK signaling pathways were reported to be activated by binding of the Gal/GalNAc-binding lectin of *Entamoeba histolytica* with the cell membrane receptor of human intestinal epithelial cell line (Henle-407) [39]. These multiple MAPK signaling pathways are known to affect the cell physiology by acting on different nuclear substrates or by binding to different transcription factors.

## Supporting Information

Figure S1 Integrin α-PS3 (A) *N*-glycosylation sites were predicted by NetNGlyc 1.0 Server. The graph illustrates predicted N-glycosylation sites across the protein chain (x-axis represents protein length from *N*- to *C*-terminal). A position with a potential (vertical lines) crossing the threshold (horizontal line at 0.5) is predicted to be glycosylated. (B) *O*-glycosylation sites were predicted by NetOGlyc 3.1 Server. A position with a potential (vertical lines) crossing the threshold (horizontal line at 0.5) is predicted to be glycosylated. (B) Prediction of the orientation in the cell membrane. The analysis was done using TMHMM 2.0 Server. Red bars and peaks represent putative transmembrane domains. Blue lines represent putative intracellular portions of the protein; pink lines represent putative extracellular portions of the protein.
(TIF)

Figure S2 Integrin β-nu (A) *N*-glycosylation sites were predicted by NetNGlyc 1.0 Server. The graph illustrates predicted *N*-glycosylation sites across the protein chain (x-axis represents protein length from *N*- to *C*-terminal). A position with a potential (vertical lines) crossing the threshold (horizontal line at 0.5) is predicted to be glycosylated. (B) *O*-Glycosylation sites were predicted by NetOGlyc 3.1 Server. A position with a potential (vertical lines) crossing the threshold (horizontal line at 0.5) is predicted to be glycosylated. (C) Prediction of the orientation in the cell membrane. The analysis was done using TMHMM 2.0 Server. Red bars and peaks represent putative transmembrane domains. Blue lines represent putative intracellular portions of the protein; pink lines represent putative extracellular portions of the protein.
(TIF)

Figure S3 Latrophilin Cirl (A) *N*-glycosylation sites were predicted by NetNGlyc 1.0 Server. The graph illustrates predicted *N*-glycosylation sites across the protein chain (x-axis represents protein length from *N*- to *C*-terminal). A position with a potential (vertical lines) crossing the threshold (horizontal line at 0.5) is predicted to be glycosylated. (B) *O*-glycosylation sites were predicted by NetOGlyc 3.1 Server. A position with a potential (vertical lines) crossing the threshold (horizontal line at 0.5) is predicted to be glycosylated. (C) Prediction of the orientation in the cell membrane. The analysis was done using TMHMM 2.0 Server. Red bars and peaks represent putative transmembrane domains. Blue lines represent putative intracellular portions of the protein; pink lines represent putative extracellular portions of the protein.
(TIF)

Figure S4 Neuroglian (A) *N*-glycosylation sites were predicted by NetNGlyc 1.0 Server. The graph illustrates predicted *N*-glycosylation sites across the protein chain (x-axis represents protein length from *N*- to *C*-terminal). A position with a potential (vertical lines) crossing the threshold (horizontal line at 0.5) is predicted to be glycosylated. (B) *O*-glycosylation sites were predicted by NetOGlyc 3.1 Server. A position with a potential (vertical lines) crossing the threshold (horizontal line at 0.5) is predicted to be glycosylated. (C) Prediction of the orientation in the cell membrane. The analysis was done using TMHMM 2.0 Server. Red bars and peaks represent putative transmembrane domains. Blue lines represent putative intracellular portions of the protein; pink lines represent putative extracellular portions of the protein.
(TIF)
