# Supplementary figures and images for: GalNAc/Gal-Binding Rhizoctonia solani Agglutinin Has Antiproliferative Activity in Drosophila melanogaster S2 Cells via MAPK and JAK/STAT Signaling
Source: PLoS One. 2012 Apr 18;7(4):e33680. doi: 10.1371/journal.pone.0033680 (PMC3329507; doi:10.1371/journal.pone.0033680)

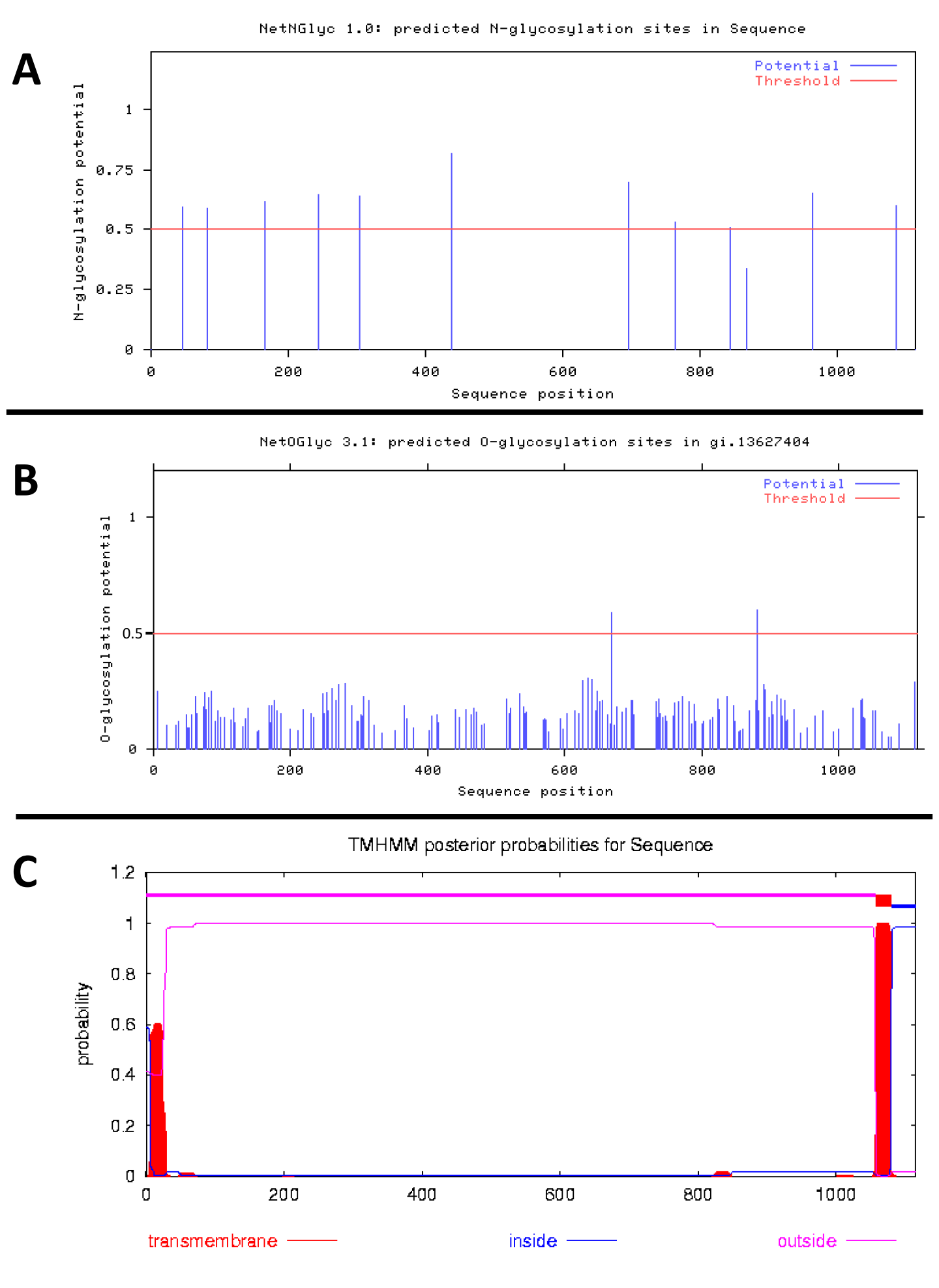

Supplement: Figure S1 — Integrin α-PS3 (A) N-glycosylation sites were predicted by NetNGlyc 1.0 Server. The graph illustrates predicted N-glycosylation sites across the protein chain (x-axis represents protein length from N- to C-terminal). A position with a potential (vertical lines) crossing the threshold (horizontal line at 0.5) is predicted to be glycosylated. (B) O-glycosylation sites were predicted by NetOGlyc 3.1 Server. A position with a potential (vertical lines) crossing the threshold (horizontal line at 0.5) is predicted to be glycosylated. (B) Prediction of the orientation in the cell membrane. The analysis was done using TMHMM 2.0 Server. Red bars and peaks represent putative transmembrane domains. Blue lines represent putative intracellular portions of the protein; pink lines represent putative extracellular portions of the protein. (TIF) [file pone.0033680.s001.tif]

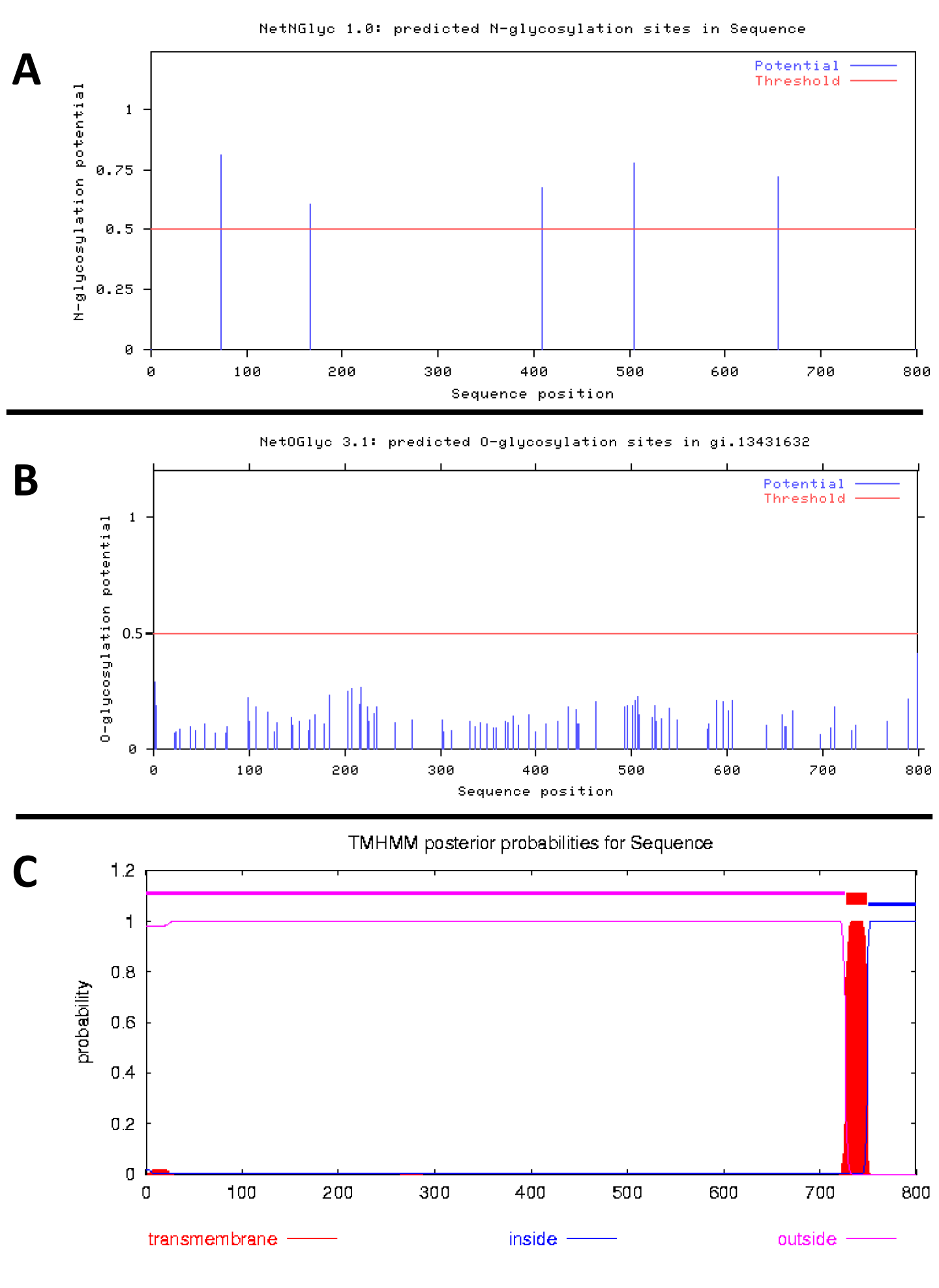

Supplement: Figure S2 — Integrin β-nu (A) N-glycosylation sites were predicted by NetNGlyc 1.0 Server. The graph illustrates predicted N-glycosylation sites across the protein chain (x-axis represents protein length from N- to C-terminal). A position with a potential (vertical lines) crossing the threshold (horizontal line at 0.5) is predicted to be glycosylated. (B) O-Glycosylation sites were predicted by NetOGlyc 3.1 Server. A position with a potential (vertical lines) crossing the threshold (horizontal line at 0.5) is predicted to be glycosylated. (C) Prediction of the orientation in the cell membrane. The analysis was done using TMHMM 2.0 Server. Red bars and peaks represent putative transmembrane domains. Blue lines represent putative intracellular portions of the protein; pink lines represent putative extracellular portions of the protein. (TIF) [file pone.0033680.s002.tif]

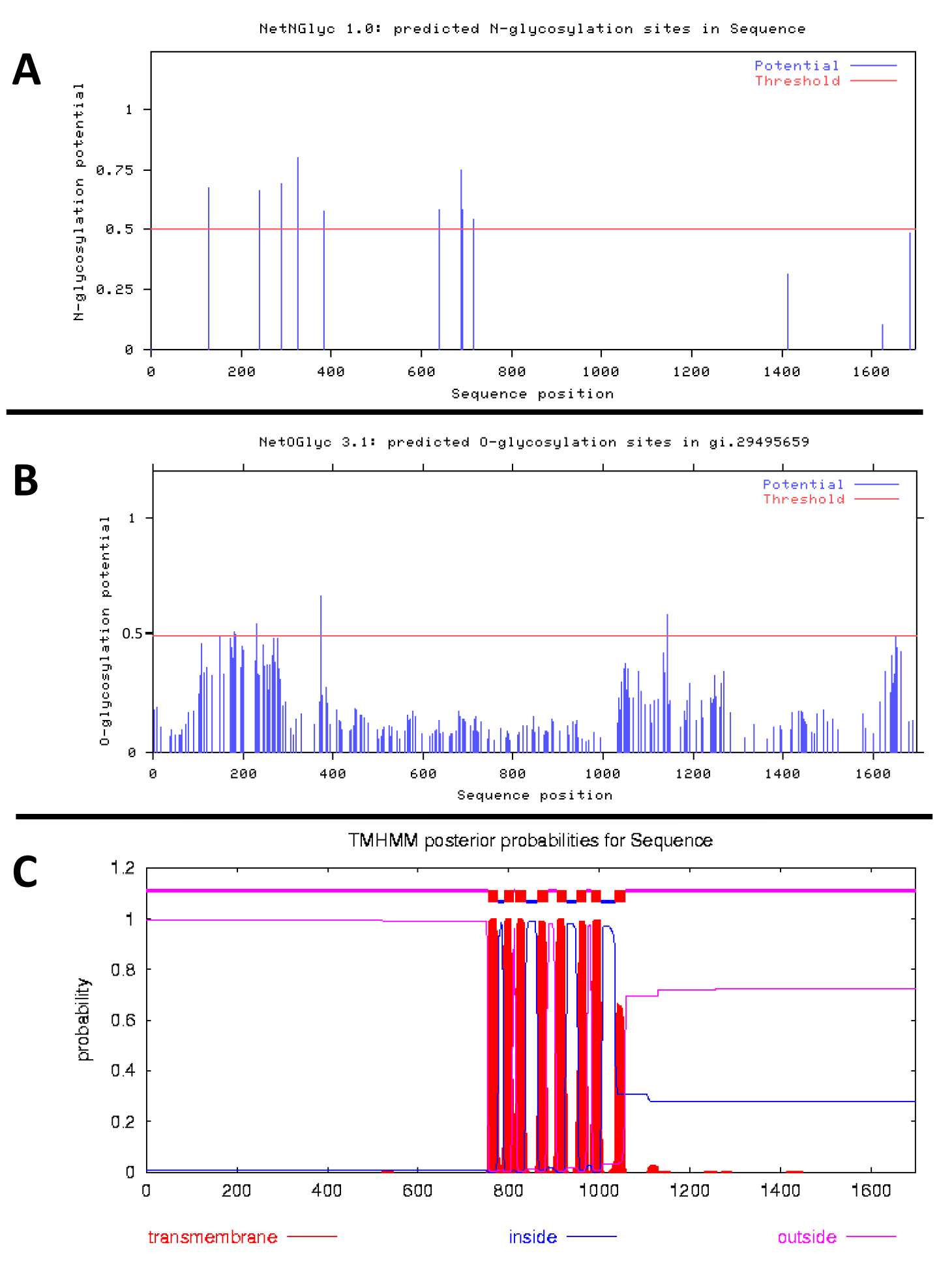

Supplement: Figure S3 — Latrophilin Cirl (A) N-glycosylation sites were predicted by NetNGlyc 1.0 Server. The graph illustrates predicted N-glycosylation sites across the protein chain (x-axis represents protein length from N- to C-terminal). A position with a potential (vertical lines) crossing the threshold (horizontal line at 0.5) is predicted to be glycosylated. (B) O-glycosylation sites were predicted by NetOGlyc 3.1 Server. A position with a potential (vertical lines) crossing the threshold (horizontal line at 0.5) is predicted to be glycosylated. (C) Prediction of the orientation in the cell membrane. The analysis was done using TMHMM 2.0 Server. Red bars and peaks represent putative transmembrane domains. Blue lines represent putative intracellular portions of the protein; pink lines represent putative extracellular portions of the protein. (TIF) [file pone.0033680.s003.tif]

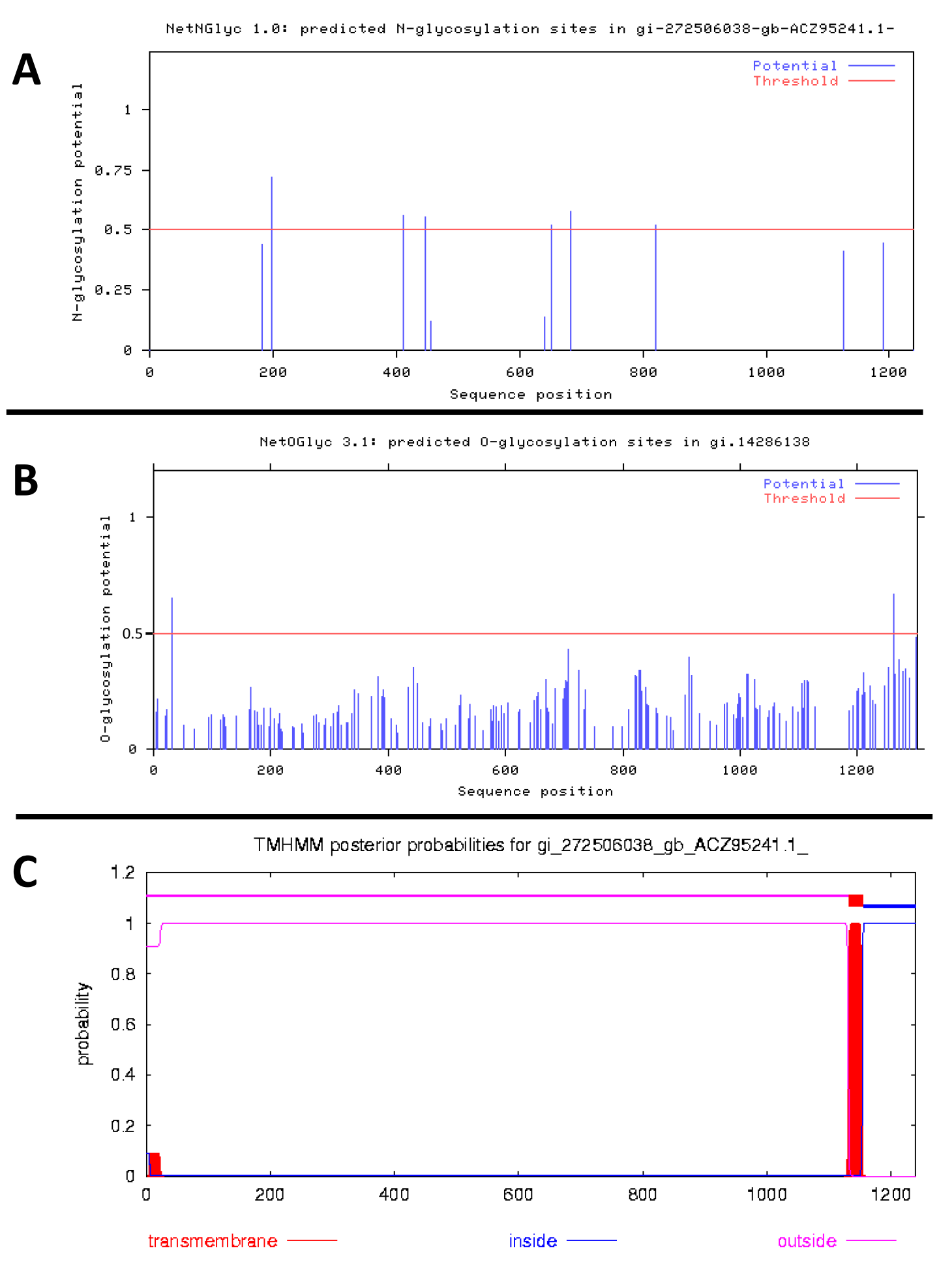

Supplement: Figure S4 — Neuroglian (A) N-glycosylation sites were predicted by NetNGlyc 1.0 Server. The graph illustrates predicted N-glycosylation sites across the protein chain (x-axis represents protein length from N- to C-terminal). A position with a potential (vertical lines) crossing the threshold (horizontal line at 0.5) is predicted to be glycosylated. (B) O-glycosylation sites were predicted by NetOGlyc 3.1 Server. A position with a potential (vertical lines) crossing the threshold (horizontal line at 0.5) is predicted to be glycosylated. (C) Prediction of the orientation in the cell membrane. The analysis was done using TMHMM 2.0 Server. Red bars and peaks represent putative transmembrane domains. Blue lines represent putative intracellular portions of the protein; pink lines represent putative extracellular portions of the protein. (TIF) [file pone.0033680.s004.tif]
